# Supplementary material for: P-Cadherin Regulates Intestinal Epithelial Cell Migration and Mucosal Repair, but Is Dispensable for Colitis Associated Colon Cancer
Source: Cells. 2022 Apr 27;11(9):1467. doi: 10.3390/cells11091467 (PMC9100778; doi:10.3390/cells11091467)
Supplement: Supplementary file 1 [file cells-11-01467-s001.zip › cells-1685440-supplementary/cells-1685440 SM for proof/P-cad supplenetry files/P-cadherin paper Table S1.pdf]

Table S1. Top 30 upregulated genes in P-cadherin knockout HCA-7 cells

GeneID	Symbol	logFC	logCPM	F	P-Value
100505920	LINC02036	10.37	1.58	702.72	1.29E-13
503639	DUXAP10	7.34	1.41	493.39	3.33E-14
357	SHROOM2	6.31	1.53	501.02	2.93E-14
503637	DUXAP8	5.86	1.70	763.59	8.28E-16
9215	LARGE1	5.78	3.27	947.20	1.32E-16
84433	CARD11	5.70	1.38	433.72	9.84E-14
23158	TBC1D9	5.54	2.33	520.80	2.24E-14
4753	NELL2	4.74	4.07	1768.39	6.22E-19
25758	KIAA1549L	4.59	4.93	815.95	1.16E-13
4008	LMO7	4.45	8.46	4964.30	8.33E-23
655	BMP7	4.05	3.46	897.73	2.08E-16
644150	WIPF3	4.01	2.41	511.69	2.45E-14
2786	GNG4	3.76	2.48	410.31	1.56E-13
10439	OLFM1	3.71	3.64	678.58	2.26E-15
10000	AKT3	3.62	5.50	2175.66	1.04E-19
130367	SGPP2	3.54	5.98	1786.38	5.70E-19
80114	BICC1	3.32	4.31	1579.86	1.64E-18
23302	WSCD1	2.91	3.01	441.37	8.50E-14
2009	EML1	2.44	4.54	662.67	2.76E-15
10810	WASF3	2.37	3.75	477.96	4.36E-14
10076	PTPRU	2.34	5.43	612.75	5.36E-15
58528	RRAGD	1.90	5.00	527.02	1.91E-14
54491	OTULINL	1.89	3.86	498.34	3.07E-14
5924	RASGRF2	1.82	4.98	454.26	6.67E-14
9619	ABCG1	1.80	4.52	547.22	1.39E-14
7128	TNFAIP3	1.75	5.91	728.12	1.24E-15
3934	LCN2	1.70	7.65	784.76	6.56E-16
22998	LIMCH1	1.62	3.88	440.86	8.58E-14
6648	SOD2	1.37	8.16	610.78	5.51E-15
2004	ELK3	1.05	7.80	399.94	1.94E-13
