# Supplementary material for: P-Cadherin Regulates Intestinal Epithelial Cell Migration and Mucosal Repair, but Is Dispensable for Colitis Associated Colon Cancer
Source: Cells. 2022 Apr 27;11(9):1467. doi: 10.3390/cells11091467 (PMC9100778; doi:10.3390/cells11091467)
Supplement: Supplementary file 1 [file cells-11-01467-s001.zip › cells-1685440-supplementary/cells-1685440 SM for proof/P-cad supplenetry files/P-cadherin paper Table S2.pdf]

Table S2. Top 30 downregulated genes in P-cadherin knockout HCA-7 cells

GeneID	Symbol	logFC	logCPM	F	P-Value
860	RUNX2	-4.37	3.66	1213.69	1.58E-17
10371	SEMA3A	-3.98	4.18	1591.38	1.54E-18
56062	KLHL4	-3.89	-0.65	163.47	2.85E-10
4069	LYZ	-3.78	7.38	2686.21	1.69E-20
10235	RASGRP2	-3.69	0.97	189.99	1.02E-10
126	ADH1C	-2.84	1.95	404.98	1.75E-13
2045	EPHA7	-2.60	2.39	585.78	7.84E-15
414332	LCN10	-2.48	1.31	174.63	1.69E-10
3075	CFH	-2.48	0.41	151.00	5.32E-10
283078	MKX	-2.46	4.29	799.04	5.63E-16
92747	BPIFB1	-2.41	2.87	334.92	8.47E-13
79365	BHLHE41	-2.11	1.84	251.37	8.97E-12
57212	TP73-AS1	-2.09	2.43	236.42	1.48E-11
53405	CLIC5	-2.03	1.92	208.00	4.17E-11
4582	MUC1	-1.95	4.38	447.33	7.59E-14
5801	PTPRR	-1.67	4.29	448.33	7.45E-14
5918	RARRES1	-1.57	3.29	245.17	1.10E-11
55130	ODAD2	-1.50	2.67	219.99	2.65E-11
3908	LAMA2	-1.48	2.72	259.79	6.86E-12
10008	KCNE3	-1.47	2.88	203.63	4.94E-11
93663	ARHGAP18	-1.45	8.94	575.30	9.13E-15
144100	PLEKHA7	-1.36	5.02	479.84	4.21E-14
1601	DAB2	-1.33	3.12	150.03	5.60E-10
3248	HPGD	-1.32	2.59	154.68	4.40E-10
23657	SLC7A11	-1.30	6.08	288.69	2.89E-12
27121	DKK4	-1.28	3.51	143.98	7.72E-10
10863	ADAM28	-1.12	3.49	172.31	1.88E-10
4047	LSS	-1.10	6.99	184.71	1.22E-10
23362	PSD3	-1.07	4.78	220.49	2.60E-11
346389	MACC1	-1.04	5.82	208.36	4.11E-11
