# Supplementary material for: P-Cadherin Regulates Intestinal Epithelial Cell Migration and Mucosal Repair, but Is Dispensable for Colitis Associated Colon Cancer
Source: Cells. 2022 Apr 27;11(9):1467. doi: 10.3390/cells11091467 (PMC9100778; doi:10.3390/cells11091467)
Supplement: Supplementary file 1 [file cells-11-01467-s001.zip › cells-1685440-supplementary/cells-1685440 SM for proof/P-cad supplenetry files/P-cadherin Revision FIgure S1 final.pptx]

## Slide 1
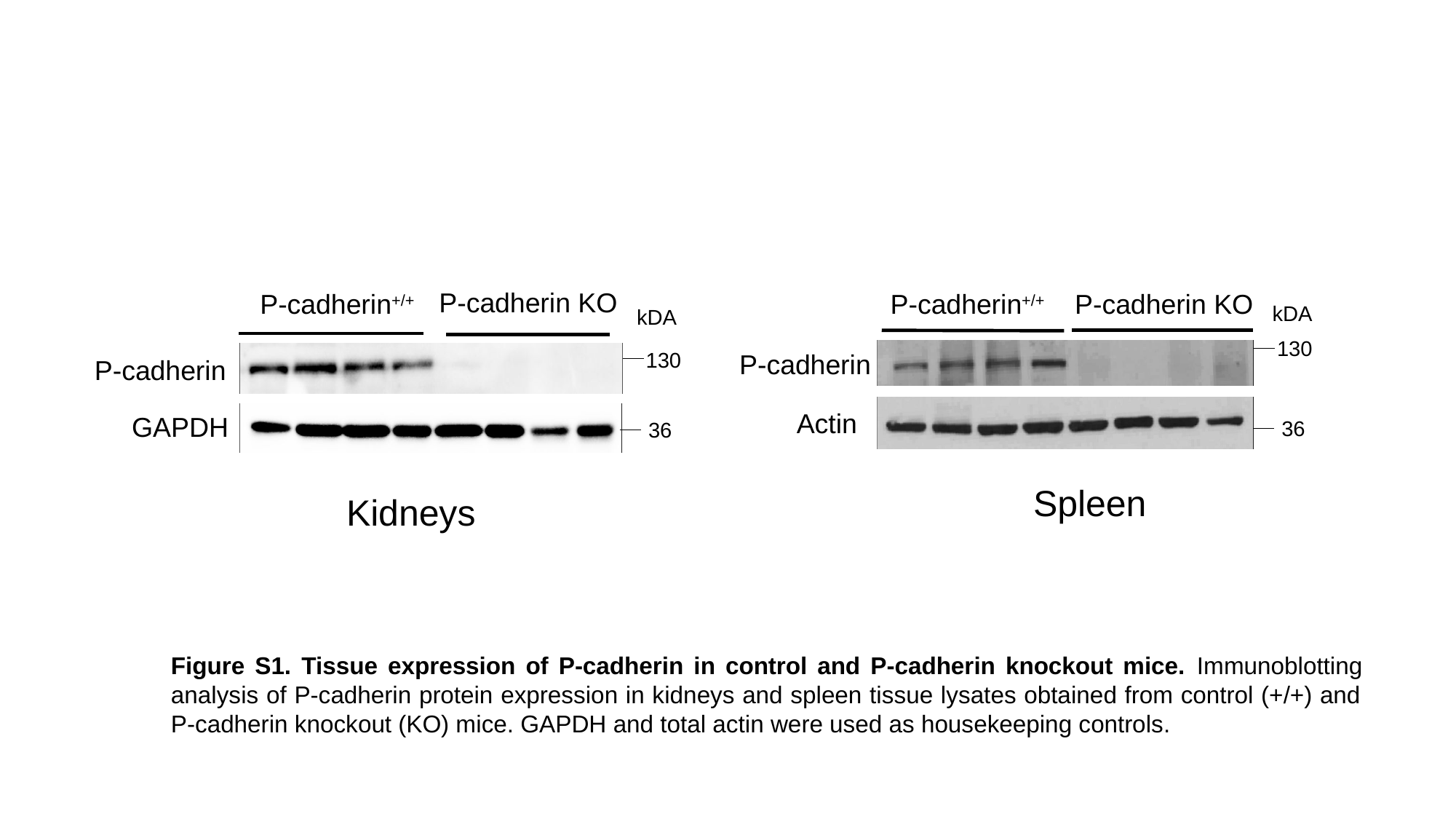

P-cadherin KO
P-cadherin+/+
P-cadherin KO
P-cadherin+/+
kDA
kDA
130
130
P-cadherin
P-cadherin
Actin
GAPDH
36
36
Spleen
Kidneys
Figure S1. Tissue expression of P-cadherin in control and P-cadherin knockout mice. Immunoblotting analysis of P-cadherin protein expression in kidneys and spleen tissue lysates obtained from control (+/+) and P-cadherin knockout (KO) mice. GAPDH and total actin were used as housekeeping controls.
