# Supplementary material for: P-Cadherin Regulates Intestinal Epithelial Cell Migration and Mucosal Repair, but Is Dispensable for Colitis Associated Colon Cancer
Source: Cells. 2022 Apr 27;11(9):1467. doi: 10.3390/cells11091467 (PMC9100778; doi:10.3390/cells11091467)
Supplement: Supplementary file 1 [file cells-11-01467-s001.zip › cells-1685440-supplementary/cells-1685440 SM for proof/P-cad supplenetry files/P-cadherin Revision Figure S2 final.pptx]

## Slide 1
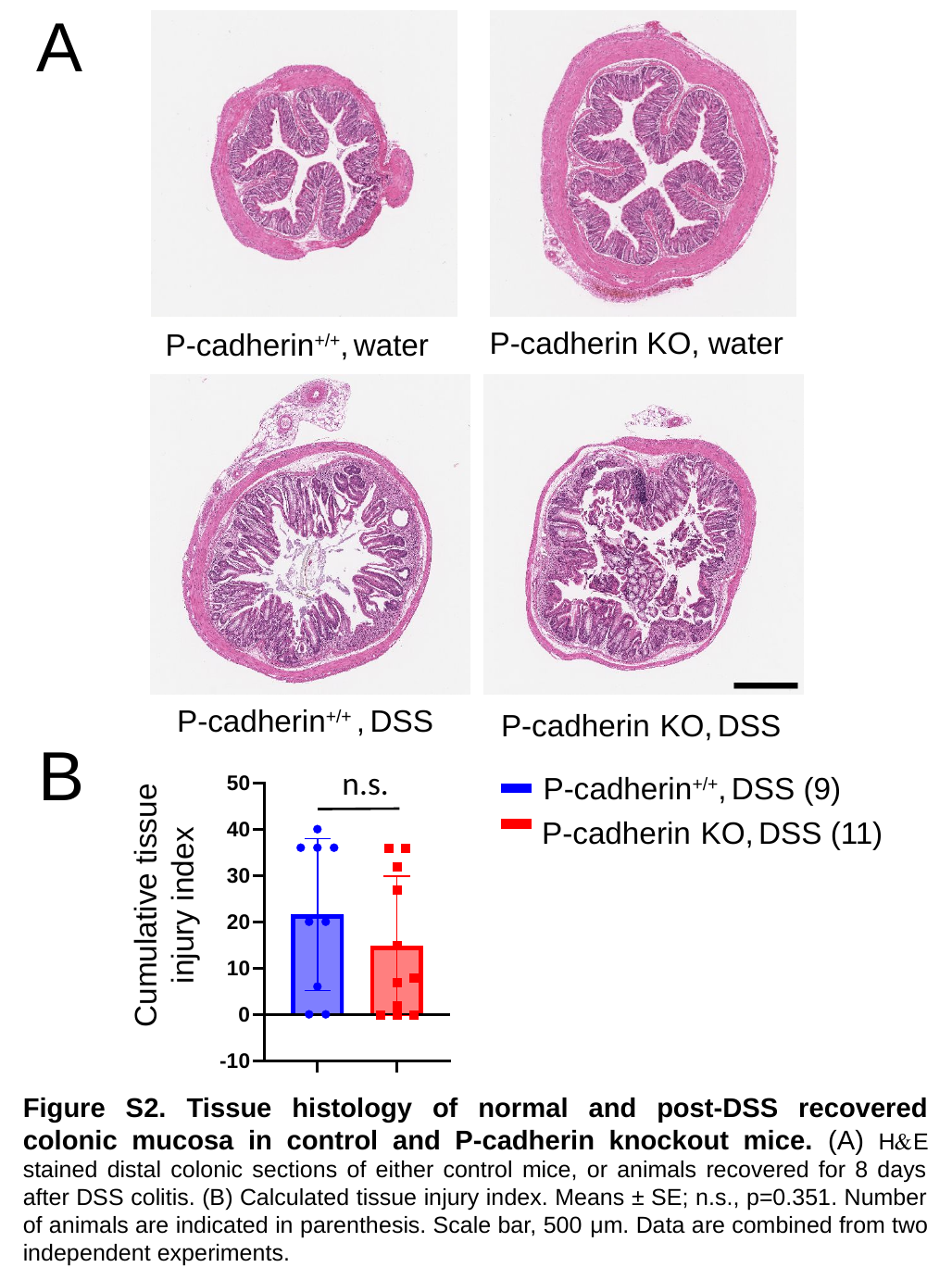

A
P-cadherin KO, water
P-cadherin+/+, water
P-cadherin+/+ , DSS
P-cadherin KO, DSS
B
n.s.
P-cadherin+/+, DSS (9)
P-cadherin KO, DSS (11)
Cumulative tissue injury index
Figure S2. Tissue histology of normal and post-DSS recovered colonic mucosa in control and P-cadherin knockout mice. (A) HE stained distal colonic sections of either control mice, or animals recovered for 8 days after DSS colitis. (B) Calculated tissue injury index. Means ± SE; n.s., p=0.351. Number of animals are indicated in parenthesis. Scale bar, 500 μm. Data are combined from two independent experiments.
