# Supplementary material for: P-Cadherin Regulates Intestinal Epithelial Cell Migration and Mucosal Repair, but Is Dispensable for Colitis Associated Colon Cancer
Source: Cells. 2022 Apr 27;11(9):1467. doi: 10.3390/cells11091467 (PMC9100778; doi:10.3390/cells11091467)
Supplement: Supplementary file 1 [file cells-11-01467-s001.zip › cells-1685440-supplementary/cells-1685440 SM for proof/P-cad supplenetry files/P-cadherin Revision FIgure S3 final.pptx]

## Slide 1
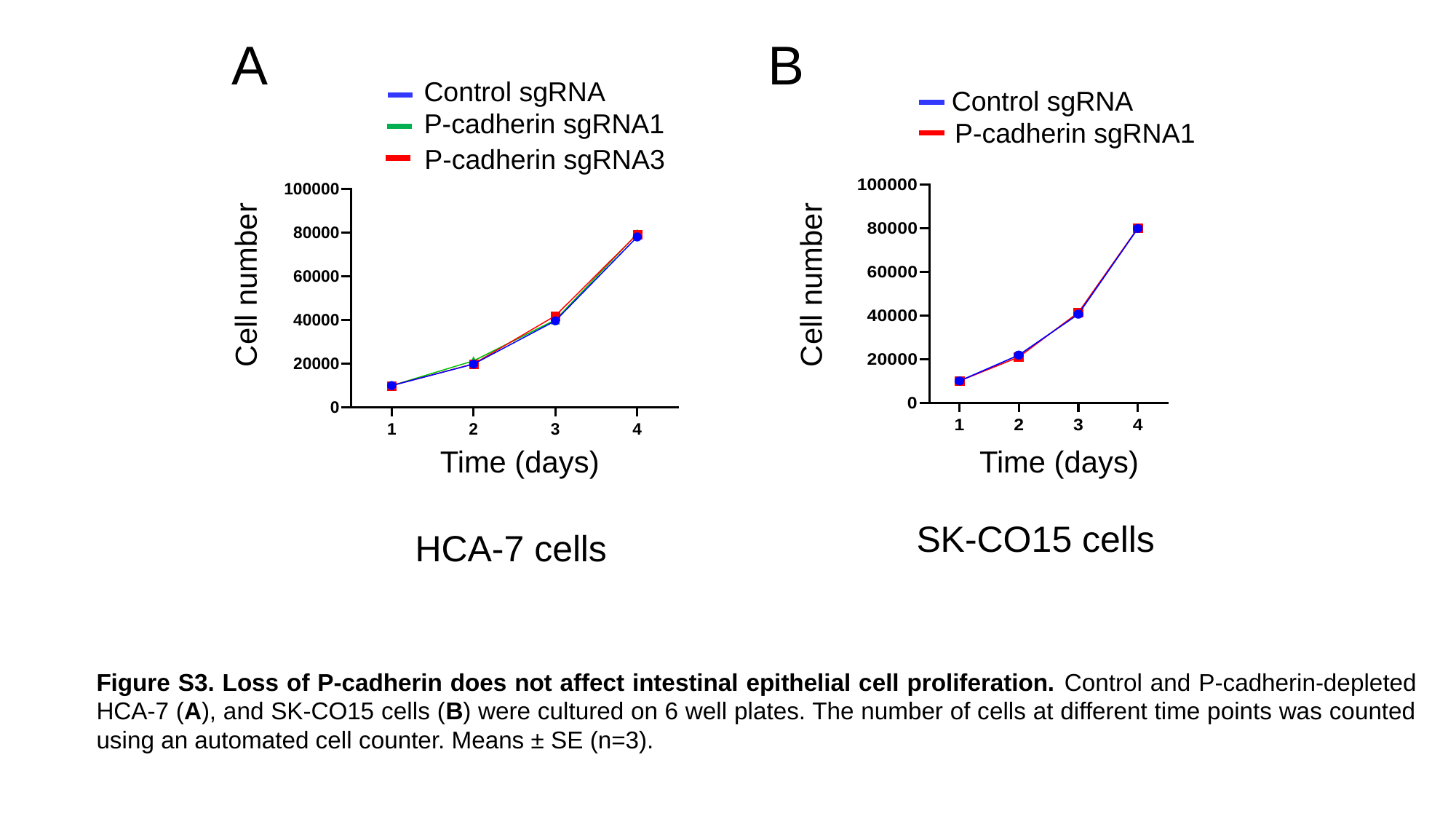

B
A
Control sgRNA
Control sgRNA
P-cadherin sgRNA1
P-cadherin sgRNA1
P-cadherin sgRNA3
Cell number
Cell number
Time (days)
Time (days)
SK-CO15 cells
HCA-7 cells
Figure S3. Loss of P-cadherin does not affect intestinal epithelial cell proliferation. Control and P-cadherin-depleted HCA-7 (A), and SK-CO15 cells (B) were cultured on 6 well plates. The number of cells at different time points was counted using an automated cell counter. Means ± SE (n=3).
