# Supplementary material for: P-Cadherin Regulates Intestinal Epithelial Cell Migration and Mucosal Repair, but Is Dispensable for Colitis Associated Colon Cancer
Source: Cells. 2022 Apr 27;11(9):1467. doi: 10.3390/cells11091467 (PMC9100778; doi:10.3390/cells11091467)
Supplement: Supplementary file 1 [file cells-11-01467-s001.zip › cells-1685440-supplementary/cells-1685440 SM for proof/P-cad supplenetry files/P-cadherin Revision Figure S4 final.pptx]

## Slide 1
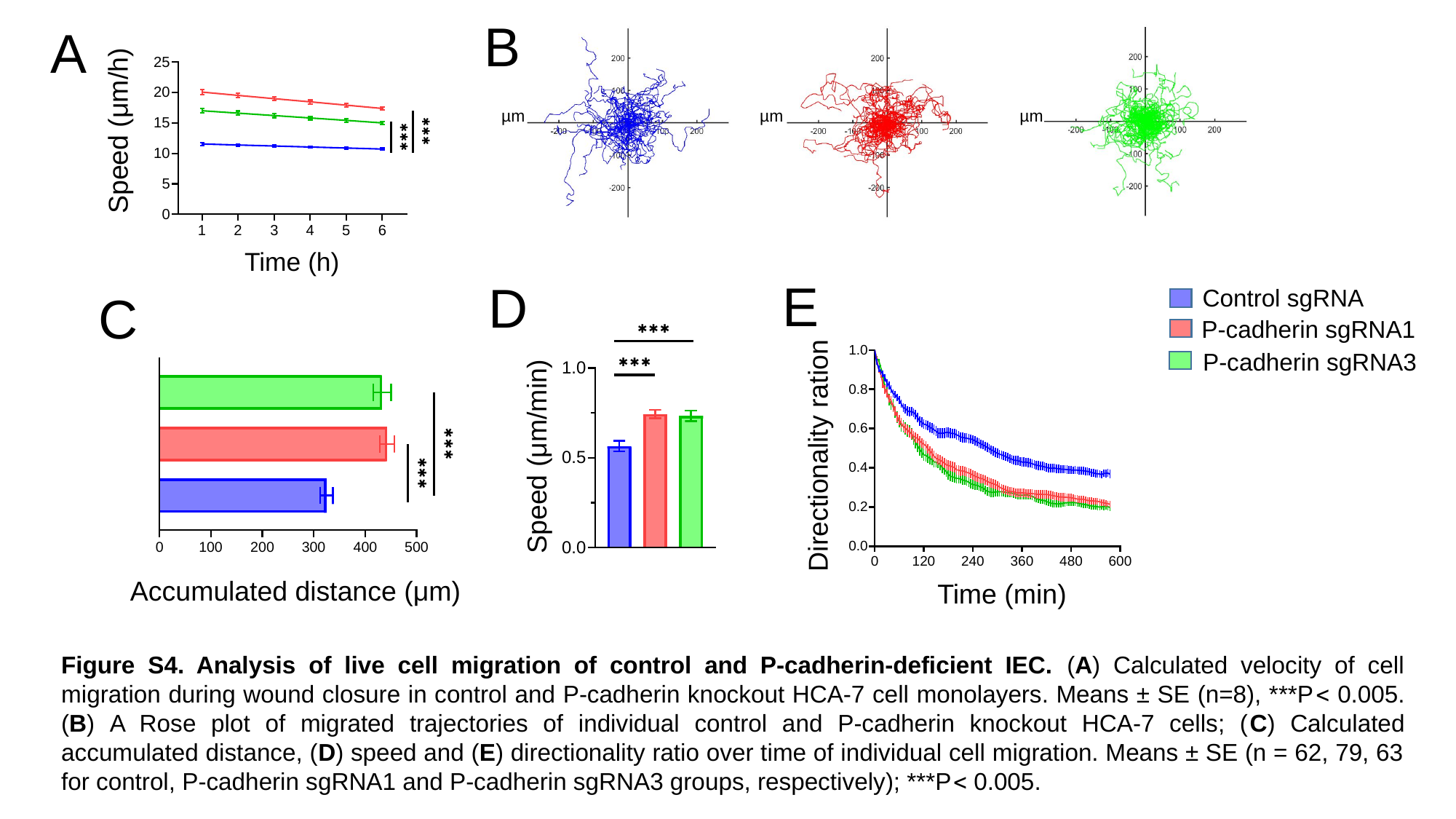

B
A
µm
µm
µm
Speed (μm/h)
Time (h)
E
D
Control sgRNA
C
P-cadherin sgRNA1
P-cadherin sgRNA3
Directionality ration
Speed (μm/min)
Accumulated distance (μm)
Time (min)
Figure S4. Analysis of live cell migration of control and P-cadherin-deficient IEC. (A) Calculated velocity of cell migration during wound closure in control and P-cadherin knockout HCA-7 cell monolayers. Means ± SE (n=8), ***P 0.005. (B) A Rose plot of migrated trajectories of individual control and P-cadherin knockout HCA-7 cells; (C) Calculated accumulated distance, (D) speed and (E) directionality ratio over time of individual cell migration. Means ± SE (n = 62, 79, 63 for control, P-cadherin sgRNA1 and P-cadherin sgRNA3 groups, respectively); ***P 0.005.
