# Supplementary material for: P-Cadherin Regulates Intestinal Epithelial Cell Migration and Mucosal Repair, but Is Dispensable for Colitis Associated Colon Cancer
Source: Cells. 2022 Apr 27;11(9):1467. doi: 10.3390/cells11091467 (PMC9100778; doi:10.3390/cells11091467)
Supplement: Supplementary file 1 [file cells-11-01467-s001.zip › cells-1685440-supplementary/cells-1685440 SM for proof/P-cad supplenetry files/P-cadherin Revision Figure S5 final.pptx]

## Slide 1
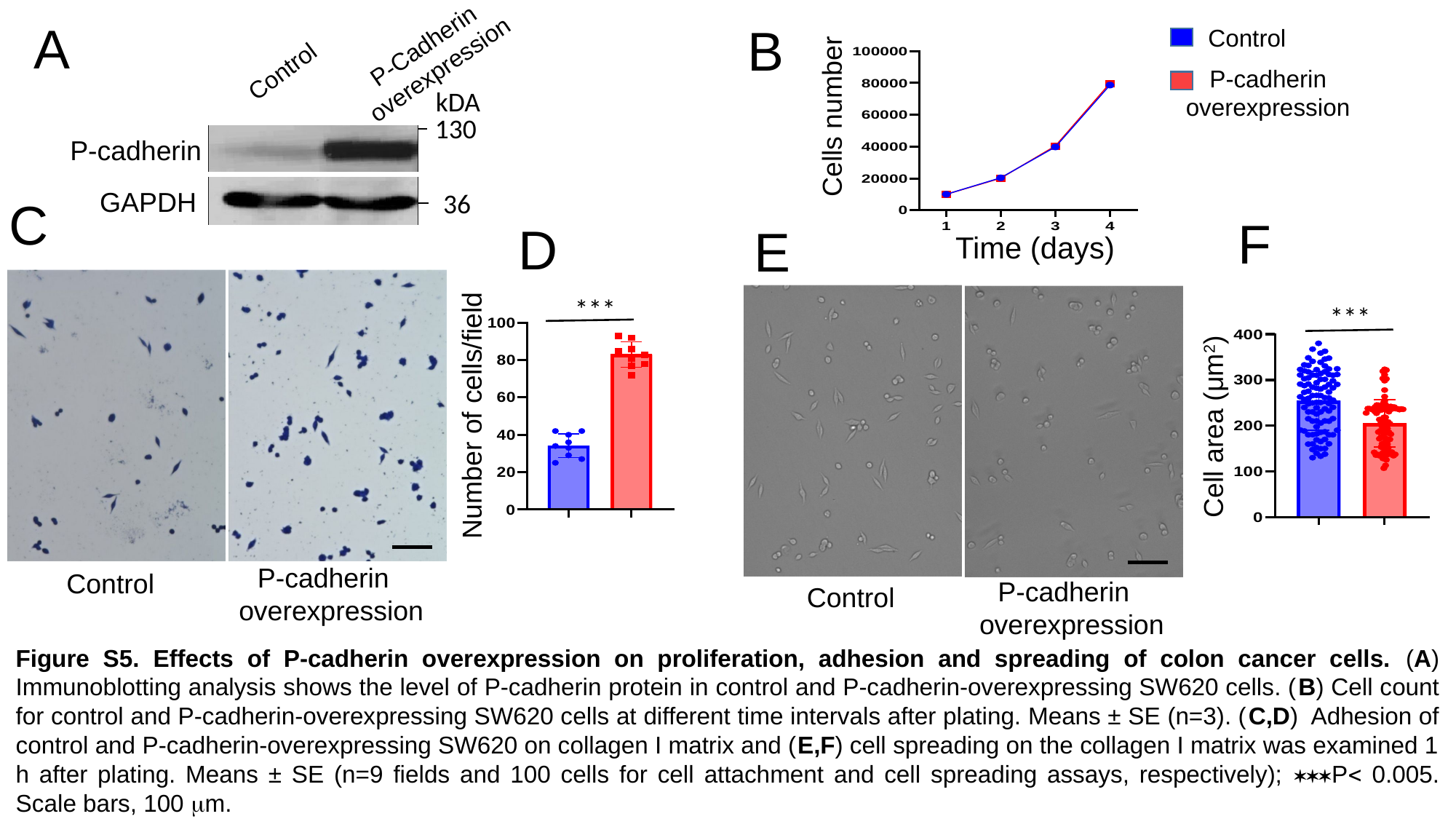

A
B
Control
P-Cadherin overexpression
Control
P-cadherin overexpression
kDA
Cells number
130
P-cadherin
GAPDH
36
C
F
D
E
Time (days)
***
***
Number of cells/field
Cell area (μm2)
 P-cadherin overexpression
Control
 P-cadherin overexpression
Control
Figure S5. Effects of P-cadherin overexpression on proliferation, adhesion and spreading of colon cancer cells. (A) Immunoblotting analysis shows the level of P-cadherin protein in control and P-cadherin-overexpressing SW620 cells. (B) Cell count for control and P-cadherin-overexpressing SW620 cells at different time intervals after plating. Means ± SE (n=3). (C,D) Adhesion of control and P-cadherin-overexpressing SW620 on collagen I matrix and (E,F) cell spreading on the collagen I matrix was examined 1 h after plating. Means ± SE (n=9 fields and 100 cells for cell attachment and cell spreading assays, respectively); P 0.005. Scale bars, 100 μm.
