# Supplementary material for: P-Cadherin Regulates Intestinal Epithelial Cell Migration and Mucosal Repair, but Is Dispensable for Colitis Associated Colon Cancer
Source: Cells. 2022 Apr 27;11(9):1467. doi: 10.3390/cells11091467 (PMC9100778; doi:10.3390/cells11091467)
Supplement: Supplementary file 1 [file cells-11-01467-s001.zip › cells-1685440-supplementary/cells-1685440 SM for proof/P-cad supplenetry files/P-cadherin Revision Figure S6 final.pptx]

## Slide 1
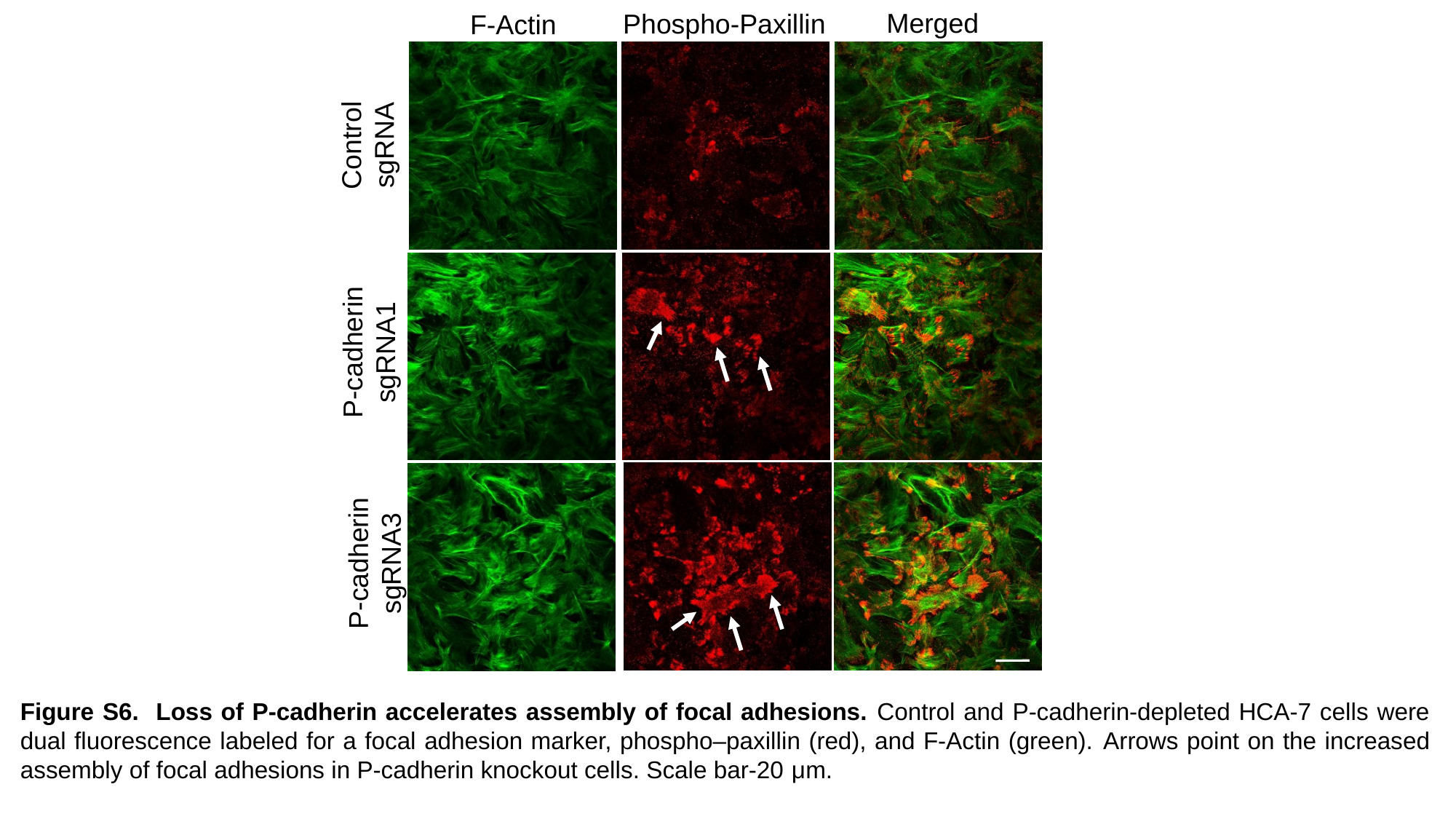

Merged
Phospho-Paxillin
F-Actin
Control sgRNA
P-cadherin sgRNA1
P-cadherin sgRNA3
Figure S6. Loss of P-cadherin accelerates assembly of focal adhesions. Control and P-cadherin-depleted HCA-7 cells were dual fluorescence labeled for a focal adhesion marker, phospho–paxillin (red), and F-Actin (green). Arrows point on the increased assembly of focal adhesions in P-cadherin knockout cells. Scale bar-20 μm.
