# Supplementary material for: P-Cadherin Regulates Intestinal Epithelial Cell Migration and Mucosal Repair, but Is Dispensable for Colitis Associated Colon Cancer
Source: Cells. 2022 Apr 27;11(9):1467. doi: 10.3390/cells11091467 (PMC9100778; doi:10.3390/cells11091467)
Supplement: Supplementary file 1 [file cells-11-01467-s001.zip › cells-1685440-supplementary/cells-1685440 SM for proof/P-cad supplenetry files/P-cadherin Revision Figure S7 final.pptx]

## Slide 1
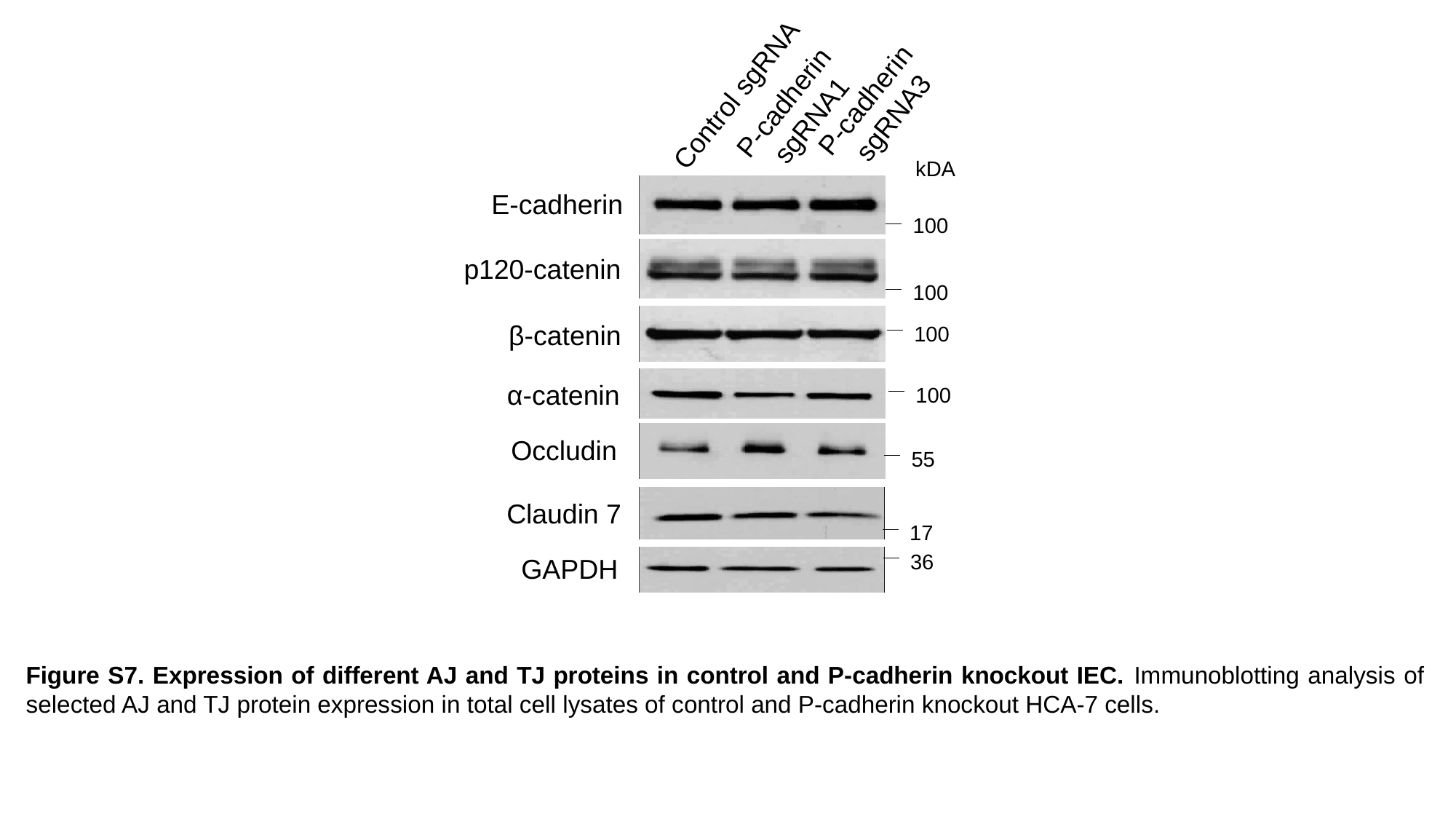

P-cadherin
 sgRNA3
Control sgRNA
P-cadherin
 sgRNA1
kDA
E-cadherin
100
p120-catenin
100
β-catenin
100
α-catenin
100
Occludin
55
Claudin 7
17
36
GAPDH
Figure S7. Expression of different AJ and TJ proteins in control and P-cadherin knockout IEC. Immunoblotting analysis of selected AJ and TJ protein expression in total cell lysates of control and P-cadherin knockout HCA-7 cells.
