# Supplementary material for: P-Cadherin Regulates Intestinal Epithelial Cell Migration and Mucosal Repair, but Is Dispensable for Colitis Associated Colon Cancer
Source: Cells. 2022 Apr 27;11(9):1467. doi: 10.3390/cells11091467 (PMC9100778; doi:10.3390/cells11091467)
Supplement: Supplementary file 1 [file cells-11-01467-s001.zip › cells-1685440-supplementary/cells-1685440 SM for proof/P-cad supplenetry files/P-cadherin Revision Figure S8 final.pptx]

## Slide 1
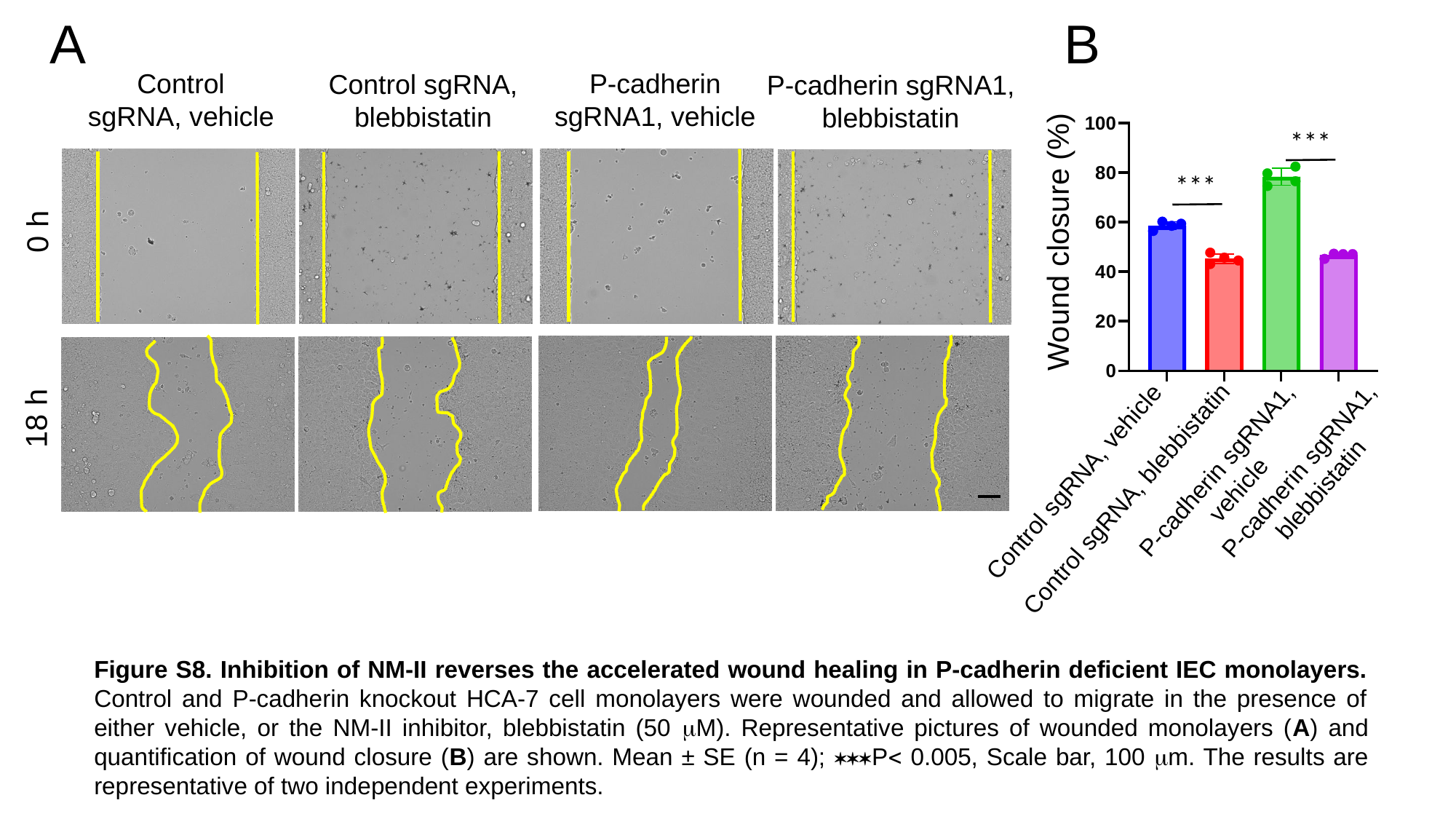

A
B
Control sgRNA, vehicle
P-cadherin sgRNA1, vehicle
Control sgRNA, blebbistatin
P-cadherin sgRNA1, blebbistatin
***
***
0 h
Wound closure (%)
18 h
P-cadherin sgRNA1, blebbistatin
P-cadherin sgRNA1, vehicle
Control sgRNA, vehicle
Control sgRNA, blebbistatin
Figure S8. Inhibition of NM-II reverses the accelerated wound healing in P-cadherin deficient IEC monolayers. Control and P-cadherin knockout HCA-7 cell monolayers were wounded and allowed to migrate in the presence of either vehicle, or the NM-II inhibitor, blebbistatin (50 μM). Representative pictures of wounded monolayers (A) and quantification of wound closure (B) are shown. Mean ± SE (n = 4); P 0.005, Scale bar, 100 μm. The results are representative of two independent experiments.
