# Supplementary figures and images for: P-Cadherin Regulates Intestinal Epithelial Cell Migration and Mucosal Repair, but Is Dispensable for Colitis Associated Colon Cancer
Source: Cells. 2022 Apr 27;11(9):1467. doi: 10.3390/cells11091467 (PMC9100778; doi:10.3390/cells11091467)

## Slide 1
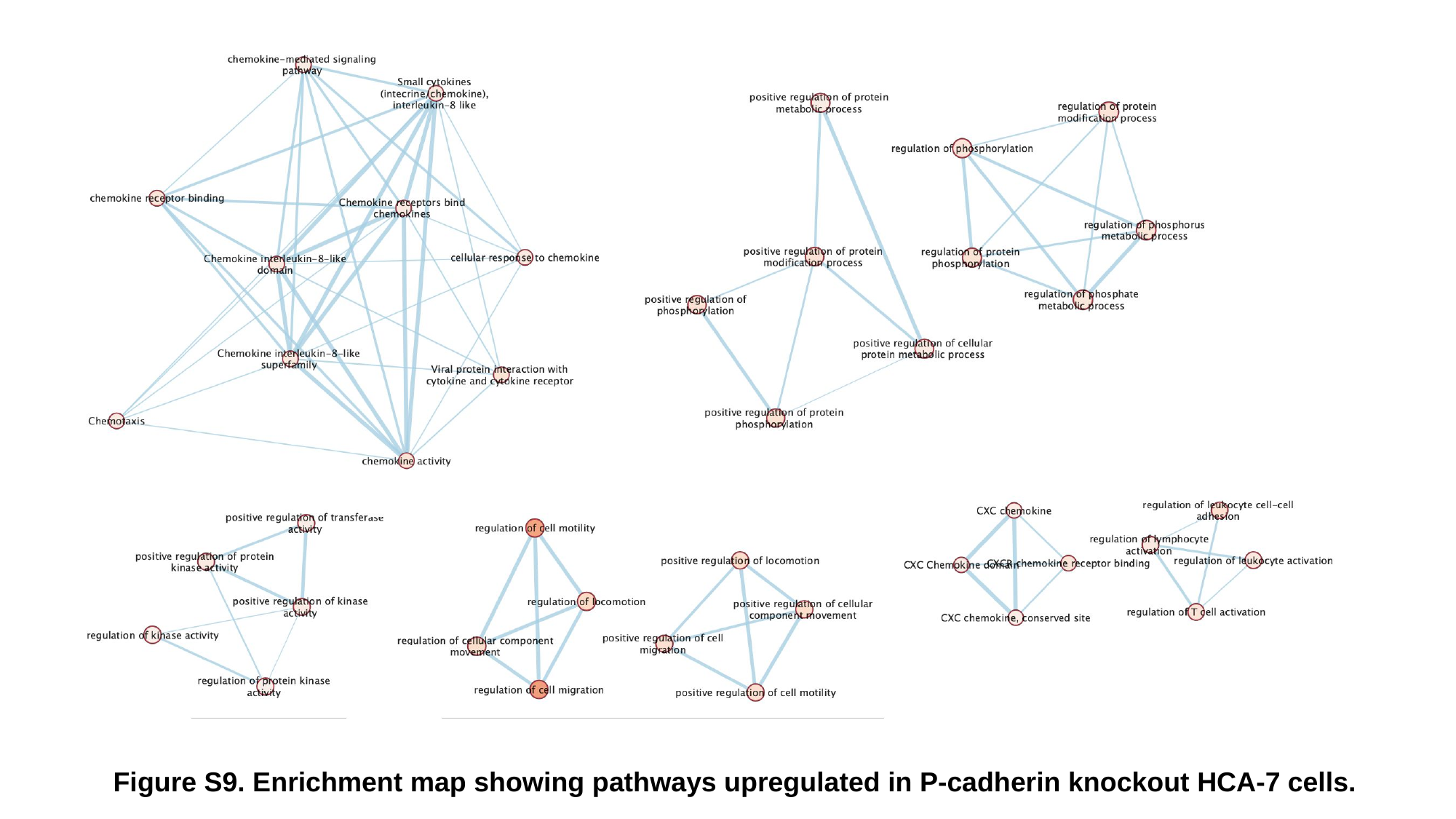

Figure S9. Enrichment map showing pathways upregulated in P-cadherin knockout HCA-7 cells.

Supplement: Supplementary file 1 [file cells-11-01467-s001.zip › cells-1685440-supplementary/cells-1685440 SM for proof/P-cad supplenetry files/P-cadherin Revision Figure S9 final.pptx]
